# Metabarcoding Close to Home: Songbird Nests as eDNA Aggregators for Trophic Ecology and Biodiversity Studies

**DOI:** 10.1002/ece3.72164

**Published:** 2025-10-13

**Authors:** John A. Kronenberger, Elise C. Zarri, Anna Noson, Taylor M. Wilcox

**Affiliations:** ^1^ National Genomics Center for Wildlife and Fish Conservation U.S. Forest Service Rocky Mountain Research Station Missoula Montana USA; ^2^ Numerical Terradynamic Simulation Group University of Montana Missoula Montana USA; ^3^ University of Montana Bird Ecology lab University of Montana Missoula Montana USA

**Keywords:** blocking oligonucleotide, environmental DNA, locked nucleic acid, sagebrush

## Abstract

Environmental DNA (eDNA) sampling to monitor wildlife presence has mostly focused on water but increasingly includes soil, air, and creative biotic substrates like flowers and spiderwebs. Biotic substrates are unique in that they also provide insight into ecological interactions. Here we explore the ability of eDNA from songbird nests to reveal avian trophic ecology, such as nestling diet and nest predator identity, in addition to local insect biodiversity. Twenty‐two nests comprising five New World sparrow species and two nonsparrow passerines were collected in a montane sagebrush steppe ecosystem shortly after confirmed nest predation events. A novel protocol was used to extract eDNA from whole nests, and each nest was sequenced twice—with and without a blocking oligonucleotide. The blocker was designed with alternating locked nucleic acids to specifically inhibit sparrow amplification and improve detection of rare species. A total of 126 species were detected, and the blocker proved highly effective, reducing sparrow reads ~100% with no discernable coblocking of nonsparrow passerines. Species richness in sparrow nests increased by 31% with the blocker when using a minimum read threshold of 10 copies. Most detected species were insects, including likely prey items and ectoparasites of nestling birds. Predators were detected in 36% of nests. We discuss the merits of this rich and unique data source and considerations for future implementation.

## Graphical abstract

We present a novel method for extracting eDNA from whole bird nests and a LNA blocking oligonucleotide designed to reduce metabarcoding reads from nest building sparrows. Metabarcoding of 22 nests from a montane sagebrush steppe ecosystem detected 126 species, primarily insects, including putative diet items, ectoparasites, and confirmed nest predators.

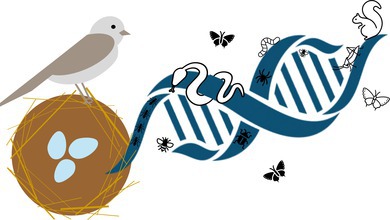

## 1 Introduction

“Earth has few secrets from the birds.”—William Beebe (1906).

Environmental DNA (eDNA) sampling is a powerful tool for wildlife monitoring that utilizes a rapidly growing suite of substrates to identify species presence. Most sampling has focused on water (Rees et al. 2014; Schenekar 2023; Takahashi et al. 2023), but soil (Hassan et al. 2022; Samuels et al. 2025; Taberlet et al. 2012) and air (Johnson and Barnes 2024) are increasingly common. Snow has shown great promise for winter carnivore monitoring (Franklin et al. 2019). Other eDNA substrates are biotic in origin or association, such as scat (Boyer et al. 2015; Nørgaard et al. 2021; Pilgrim et al. 2023), invertebrate bloodmeals (sometimes called iDNA; Calvignac‐Spencer et al. 2013; Lynggaard et al. 2022), marine sponges (Mariani et al. 2019), ants (Lin et al. 2025), spiderwebs (Gregorič et al. 2022), and the surfaces of flowers (Johnson et al. 2023; Thomsen and Sigsgaard 2019) and other vegetation (Coetzer 2024; Valentin et al. 2020), including “rainwash” dripping from forest canopies (Macher et al. 2023).

Biotic eDNA substrates are unique in that detections can be associated with the ecology of the source organism. Many organisms actively influence eDNA aggregation through behavior (scat, bloodmeals, spiderwebs) or attraction (flowers) and therefore provide a snapshot of trophic interactions in addition to local biodiversity data, resolving taxa that may be underrepresented by more passive biotic substrates (leaves, trunks) and those that are principally abiotic (water, soil, air, snow). Indeed, many studies have espoused the idea of organisms as eDNA samplers (including Boyer et al. 2015; Calvignac‐Spencer et al. 2013; Gregorič et al. 2022; Johnson et al. 2023; Lynggaard et al. 2022; Mariani et al. 2019; Nørgaard et al. 2021; Thomsen and Sigsgaard 2019). Recently, Levesque‐Beaudin et al. (2023) demonstrated the use of songbird nests as a biotic substrate for eDNA sampling, providing a unique perspective into avian ecology during the breeding season.

Songbird nests are epicenters for a host of interesting ecological interactions, including nestling diet, parasitism, and predation. However, these interactions have remained notoriously difficult to study. For example, identifying prey items brought to the nest can be laborious, invasive, or taxonomically coarse. Previous studies have used visual observation in the field with binoculars or nest cameras (Hoenig et al. 2022), metabarcoding of feces (Bookwalter et al. 2023; Cabodevilla et al. 2024; Spence et al. 2022) or swabs of the digestive tract (Elmore et al. 2024), and even nestling neck ligatures (Robinson et al. 2010). Other interactions at the nest can be even more difficult to characterize. Avian ectoparasites require great expertise to identify morphologically with much precision (Mathison and Pritt 2014) and identification of endoparasites requires blood or feces collection and subsequent microscopic or genetic analysis (Bourret et al. 2021). Nest predator identification is particularly difficult and continues to be a major missing piece in avian nesting studies (Ibáñez‐Álamo et al. 2015; Weidinger 2008). Often, the potential suite of nest predators is vast, and identification relies on laborious video monitoring that is intrusive and potentially impactful to predator behavior (Ibáñez‐Álamo et al. 2015; Richardson et al. 2009; Schroeder et al. 2025), although recent advances have been made through genetic identification via eggshell swabs and hairs left at depredated nests (Helmstetter et al. 2024; Hopken et al. 2016; Kirol et al. 2018). Nest metabarcoding has the potential to illuminate all of these interactions (i.e., nestling diet, parasites, and predation) while also providing unique biodiversity information to complement tallies from other survey methods such as identification of bulk insect samples from malaise, light, pan, or pitfall traps and acoustic monitoring (Montgomery et al. 2021).

Here, we demonstrate metabarcoding of songbird nests and its utility for two applications: (1) revealing avian trophic interactions and (2) insect biodiversity surveys. To accomplish this, we present a novel whole‐nest eDNA extraction method and a blocking oligonucleotide designed with alternating locked nucleic acids to specifically limit New World sparrow (Passerellidae) amplification and increase detection of rare species. Furthermore, each nest in this study was recently depredated prior to collection, often with predator identity confirmed on video. We take advantage of this uncommon and valuable information to assess the feasibility of using nest metabarcoding to investigate nest predation events.

## 2 Methods

### 2.1 Study Site

**FIGURE 1:**
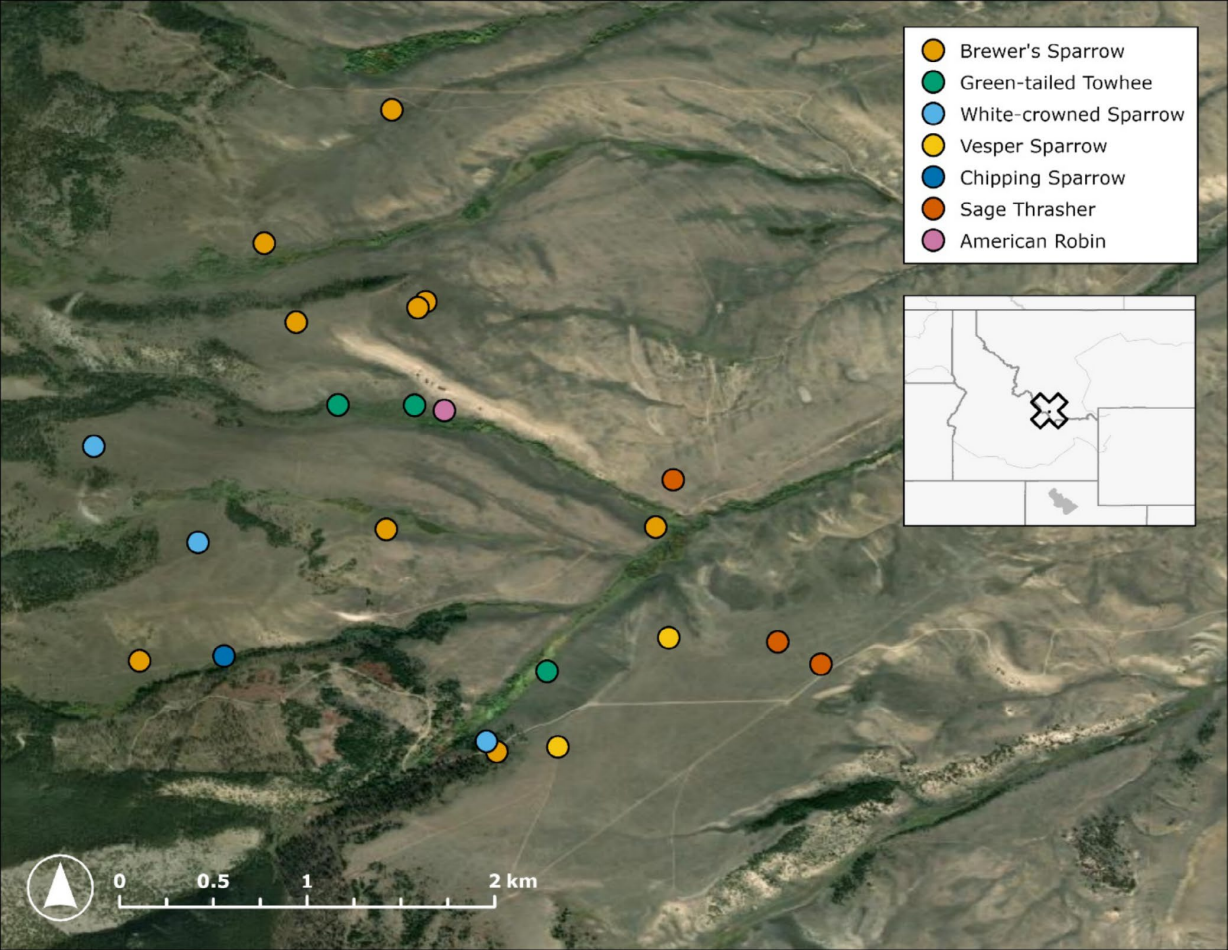
Map of nest locations in the Medicine Lodge Valley of southwest Montana.

The field component of this study was conducted in the Medicine Lodge Valley of southwest Montana at the ecotone between conifer forest and sagebrush habitats, representing a montane sagebrush steppe ecosystem (Figure 1). Vegetation consisted primarily of mountain big sagebrush (*Artemisia tridentata vaseyana*), Douglas fir ( *Pseudotsuga menziesii* ), and juniper (*Juniperus* spp.).

### 2.2 Nest Monitoring and Collection

Nests were found within an area of ~4 km^2^ through observations of parental behavior and systematic searching as part of a larger study (Zarri et al. 2024). Nests were checked every 1 to 3 days to record contents and fate. We randomly selected nests to be filmed with a camouflaged video camera (Cannon Vixia HF R800) throughout the breeding seasons (May–August) of 2020 to 2022 to record parental behavior such as feeding rate and time spent brooding. Filming occurred for 4 to 12 h per session, and cameras were collected the following day. When nest contents disappeared between camera set‐up and take‐down, we reviewed the footage to confirm nest predation. Other predation events were confirmed through direct observation or indirect evidence during monitoring, such as missing or partially consumed nestlings. Depredated nests were collected within an average of 4 days (range 0–16; Data S1) using nitrile gloves, placed in zip‐top plastic bags, and frozen within 8 h of collection. Nest monitoring and collection were conducted under Montana Fish Wildlife and Parks permits 2019‐010‐W, 2020‐008‐W, 2021‐021‐W, and 2022–033‐W, U.S. Fish and Wildlife Service permit MB791101‐0, U.S. Geological Survey federal bird banding permit 21,635, and University of Montana Institutional Animal Care and Use Committee Animal Use Proposals 061‐18 and 046‐20.

### 2.3 eDNA Extraction

We extracted eDNA from bird nests by combining elements of a large‐volume soil eDNA extraction method (Taberlet et al. 2012) with buffer filtration, similar to the process described by Samuels et al. (2025). Briefly, each nest was placed in a 2 L roll‐top plastic bag, to which sodium phosphate buffer (0.12 mM, pH ~8) was added at a volume needed to almost fully immerse the nest (500–2000 mL; Data S1). The nest‐buffer mixture was shaken manually every 5 min for 20 min. Then, the mixture was poured through a paper towel to remove debris and filtered using a 0.2 μm pore size, 47 mm diameter nitrocellulose filter (Nalgene) and peristaltic pump (Geotech) until clogging (25–225 mL). Filters were stored in silica desiccant and frozen at −20°C until extraction. DNA was extracted from entire filters using the modified DNeasy Blood and Tissue Kit (QIAGEN) protocol of Franklin et al. (2019) and treated with a PCR inhibitor removal kit (Zymo). All work was conducted in sterile, pre‐PCR laboratory spaces and extractions occurred under a hood after irradiation with UV light for 1 h.

### 2.4 Primer Selection and Blocker Design

We chose the BF1 and BR2 primers developed by Elbrecht and Leese (2017), which amplify a 356 bp region of the mitochondrial cytochrome c oxidase subunit I (*COI*) gene and largely match other commonly used animal primers (Folmer et al. 1994; Geller et al. 2013; Leray et al. 2013). To ensure their utility in our study, we used the *rentrez* package (Winter 2017) and a custom script in *R* (version 4.0.4; R Core Team 2023) to download *COI* sequences from GenBank (Sayers et al. 2023), including 133 (69%; all available) of 193 vertebrate species common in the montane sagebrush steppe ecosystem, according to the Montana Natural Heritage Program (2024). Sequences were aligned with primers using the *MUSCLE* (Edgar 2004) algorithm in *MEGA* (version 11; Tamura et al. 2021). We modified the BF1 primer at three bases to provide a better match while maintaining a similar level of degeneracy. Final primer sequences (with linker sequences and modified bases underlined) were BF1_alt_tailed: GTGACTGGAGTTCAGACGTGTGCTCTTCCGATCTACAGGHTGRACHGTNTAYCC and BR2_tailed: ACACTCTTTCCCTACACGACGCTCTTCCGATCTTCDGGRTGNCCRAARAAYCA. Primer generality was assessed *in silico* using the “primer‐only” version of *eDNAssay*—a random forest classifier trained to predict amplification with high accuracy (Kronenberger et al. 2022). For each sequence, the classifier outputs an assignment probability (AP), or the proportion of decision trees choosing the “amplify” class. Prior experience indicates that the likelihood of amplification is very high at an AP of 0.7 or greater. To help put APs into context, we also report total mismatches and those on the 3′ end (first five bases), which are most impactful (Wright et al. 2014).

In their study of nest eDNA, Levesque‐Beaudin et al. (2023) found that the large majority of reads were from the nesting songbird itself. We therefore designed a blocking oligonucleotide to minimize amplification of New World sparrows (Passerellidae; comprising 18 of 22 nests) and increase reads for rare animal templates. To do so, we downloaded *COI* sequences from 65 additional New World sparrow species (Data S1) and aligned them with the sequences described above. We positioned the blocker in a region between primers that appeared to be relatively conserved for sparrows and divergent from other species. Blocking capacity was maximized by making every other base a locked nucleic acid (LNA), with mismatches concentrated in the center for greater impact as described by Prout et al. (2023). We adjusted blocker length for a melting temperature ~7°C higher than that of primers to encourage earlier annealing. The final sequence (with 5′ and 3′ modifications and a plus sign before each LNA) was /5AmMC6/C + AG + TY + GA + CC + TY + GC + AA+T/3AmMO/. Performance was assessed *in silico* using the “primer‐and‐probe” version of *eDNAssay* (Kronenberger et al. 2022, 2024) to count total mismatches and those in the center (more than five bases from either end). To do this, the blocker was positioned in the third (probe) row of the alignment and APs were disregarded.

### 2.5 Metabarcoding

Each nest was sequenced with and without the sparrow blocker to evaluate its impact. We used a two‐step PCR protocol—first producing target amplicons using primers tailed with linkers for hybridization in the second PCR, which functions to add unique indexes and sequencing adapters. Target amplification reactions were run in quadruplicate and pooled to ameliorate stochasticity in the amplification of rare templates. Reactions were 12 μL and contained 2 μL template DNA, 1× reaction buffer (Life Technologies), 2.5 mM MgCl_2_, 200 μM each dNTP, 1 μM each primer, and 0.4 U AmpliTaq Gold polymerase (Life Technologies). Sparrow blocking reactions also included 1 μM blocking oligonucleotide. We used a touchdown thermal cycling profile to reduce nontarget amplification and dimer formation as follows: 95°C/3 min, (98°C/20 s, 65°C/1 min [−1°C/cycle], 72°C/20 s) × 10 cycles, (98°C/20 s, 55°C/1 min, 72°C/20 s) × 40 cycles, 72°C/5 min. During all stages of library preparation, we included a positive control containing genomic DNA from an American eel ( *Anguilla rostrata* ), which does not occur in the area, to assess contamination risk. Reaction products were evaluated for quality and quantity using 1.6% agarose gel electrophoresis, cleaned using 0.9× Ampure XP beads (Beckman Coulter), and eluted to 20 μL in Tris‐EDTA buffer.

Indexing reactions were 25 μL and contained 3 μL cleaned PCR product, 500 nM each of Illumina P5 and P7 adaptors with unique dual indexes (as in Meyer and Kircher 2010), and 1× KAPA HiFi HotStart ReadyMix (Roche). Each index was used only once to minimize the impact of index switching. The thermal cycling profile was 98°C/30 s, (98°C/10 s, 60°C/20 s, 72°C/20 s) × 12 cycles, 72°C/5 min. Reaction products were bead‐cleaned as described above, quantified using a Qubit Flex fluorometer, pooled in equimolar ratios (80 ng each), then bead‐cleaned again. The pooled, cleaned library was quantified using a Qubit Flex fluorometer, checked for quantity and quality using a TapeStation 4200 system (Agilent), and sequenced by the University of Montana Genomics Core using one lane of an Illumina MiSeq v2 kit with 2 × 250 cycles.

### 2.6 Bioinformatics

Sequencing results were demultiplexed using *bcl2fastq* from Illumina and further processed using the *R* package *DADA2* (version 1.16; Callahan et al. 2016). Specifically, demultiplexed reads were trimmed to remove the first 20 bp on the 5′ end, corresponding to primer sequences. Reads were then filtered to remove sequences that (1) contained ambiguous bases, (2) were under 25 bp in length, (3) were from the PhiX control library included in the Illumina MiSeq run, and (4) had more than two expected errors per read given quality score estimates. A sequencing error model was trained using pooled samples and quality score estimates ascribed to each possible nucleotide transition. Reads were dereplicated to infer amplicon sequence variants (ASVs) and sample inference was performed, conditional on the error model, using “pseudo‐pooled” samples. To increase sensitivity to very rare ASVs (e.g., singletons), sample inference included sequences from montane sagebrush steppe vertebrates as priors. Forward and reverse reads were merged and removed of chimeric sequences. Taxonomy was assigned to each ASV using the *RDP Classifier* (Wang et al. 2007) and all unique haplotypes of every species in the *MIDORI2* database of quality controlled eukaryotic *COI* sequences (Leray et al. 2022) via its public server (Leray et al. 2018), based on GenBank release 248 from February 2022. Taxonomy classified with at least 80% confidence was deemed legitimate. To gain insight into unassigned ASVs, we also conducted a *BLAST* search (Camacho et al. 2009) of GenBank sequences as described by Vanderpool et al. (2024), then pulled taxonomic identifiers for top hits in *R* using the *rentrez* package and assigned taxonomy to the Global Biodiversity Information Facility database using the *taxize* package (Chamberlain et al. 2020).

### 2.7 Statistics

Sequencing results produced with and without the sparrow blocker were pooled for each nest when calculating summary statistics, except for some evaluations of the blocker and minimum read thresholds. In these cases, which are noted in the text, results were either analyzed on a per‐nest, per‐songbird, or site‐wide basis (i.e., total reads regardless of source). We tested for significant differences in mean species richness per nest with and without the sparrow blocker via paired *t*‐tests using the *R* package *stats* (base package). Dietary differences among songbirds were assessed via nonmetric multidimensional scaling (NMDS) applied to read counts of ASVs within likely prey orders using the *R* package *vegan* (Oksanen et al. 2025). Specifically, we used a Bray–Curtis dissimilarity index and three dimensions, then estimated goodness of fit (stress) and conducted a permutational multivariate analysis of variance (PERMANOVA) to test whether the identity of likely prey items causes songbird species to differ in spread or position within multivariate space. We also used *vegan* to generate species accumulation curves for each analysis—with and without the blocker and pooled—through 100 random permutations of the data (subsampling without replacement), an indication of the potential for continued new detections with sequencing of additional nests.

## 3 Results

We collected 22 depredated nests from seven songbird species: Brewer's Sparrow ( *Spizella breweri* , *n* = 9), Green‐tailed Towhee ( *Pipilo chlorurus* , *n* = 3), White‐crowned Sparrow ( *Zonotrichia leucophrys* , *n* = 3), Vesper Sparrow ( *Pooecetes gramineus* , *n* = 2), Chipping Sparrow ( *Spizella passerina* , *n* = 1), Sage Thrasher ( *Oreoscoptes montanus* , *n* = 3) and American Robin ( *Turdus migratorius* , *n* = 1). All species are sparrows except for Sage Thrasher and American Robin.

Metabarcoding returned 10,000,073 reads from 11,161 ASVs. Taxonomic assignment to the *MIDORI2* database occurred with ≥ 80% confidence for 221 ASVs representing 126 distinct species, all of which were animals. However—as may be expected with environmental samples—evaluation of top *BLAST* hits revealed that the majority of reads were, in fact, nontarget (i.e., nonanimal). Excluding one Brewer's Sparrow nest that had unusually few reads without the sparrow blocker due to an indexing issue, animals accounted for 34% of reads in the no‐blocker analysis, versus 56% for fungi and 6% for bacteria; the remaining 4% were either unassigned or assigned to other kingdoms (Figure S1). The species with the most reads overall (20% of total) was *Sydowia polyspora*, a fungal pathogen of plants including Douglas fir, the dominant tree in the area. No reads were detected for the positive control (American eel), suggesting little to no contamination during library preparation.

**FIGURE 2:**
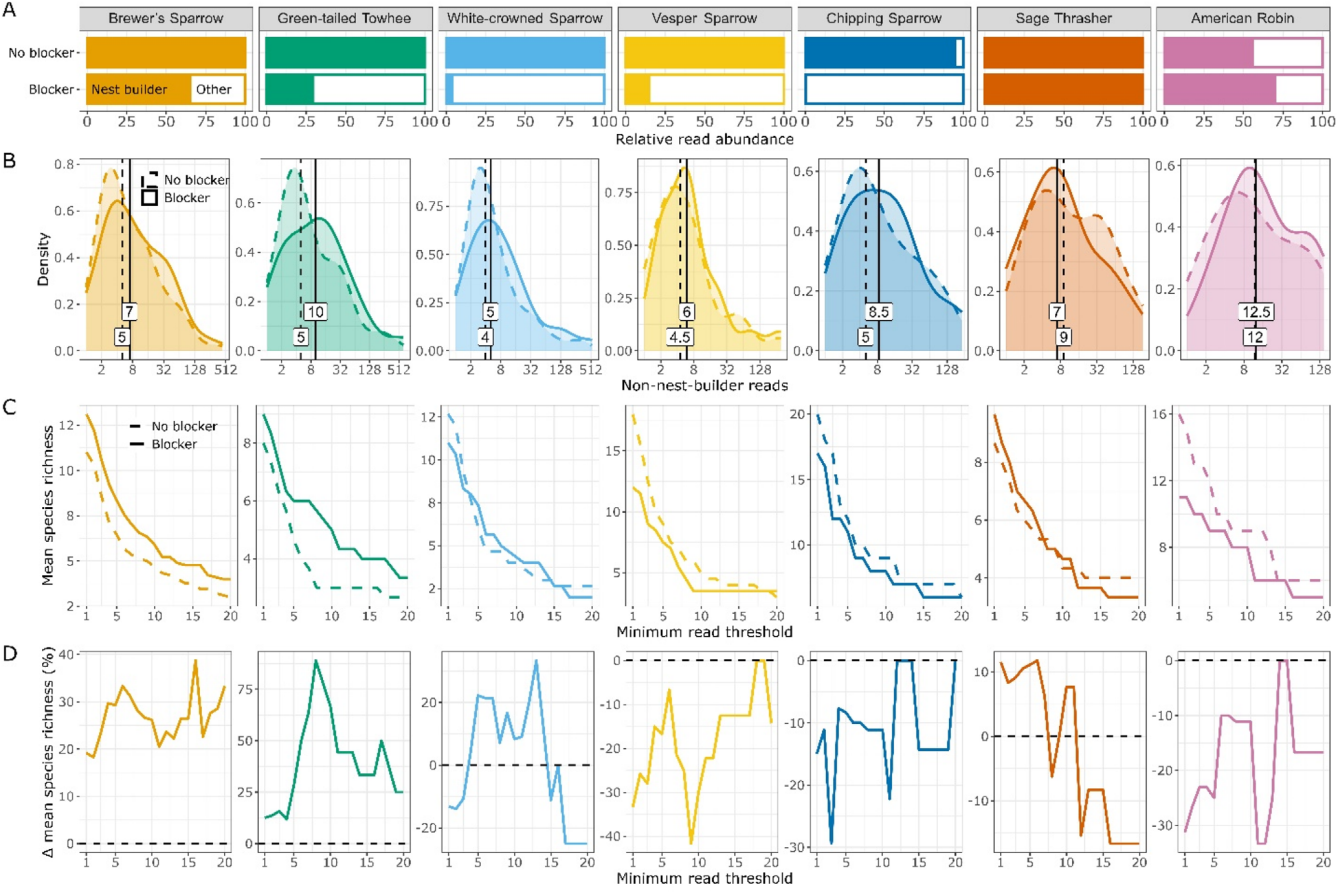
Impacts of the sparrow blocker on reads that were taxonomically assigned to the *MIDORI2* reference database, including the proportion of reads assigned to nest building songbirds versus other animal species (A); the density of read counts from nonsparrow animals, with vertical lines and numbers indicating medians (B); the mean number of species detected per nest for a range of minimum read thresholds (C); and the percent change in mean species richness when the blocker was used, with the dotted line at 0 indicating no difference (D). All nest builders are sparrows except for Sage Thrasher and American Robin. The number of nests per species varies: Brewer's Sparrow, *n* = 8 (one excluded due to unusually low read abundance without the sparrow blocker, attributed to an indexing issue); Green‐tailed Towhee, *n* = 3; White‐crowned Sparrow, *n* = 3; Vesper Sparrow, *n* = 2; Chipping Sparrow, *n* = 1; Sage Thrasher, *n* = 3; American Robin, *n* = 1.

We sequenced each nest twice—with and without a blocking oligonucleotide designed with alternating locked nucleic acids to specifically inhibit New World Sparrow amplification. Nesting sparrows produced a total of 1,189,276 reads without the blocker and 5108 reads with the blocker (~100% blocking efficiency). The blocker was also specific to sparrows. For example, songbirds produced nearly all reads that were taxonomically assigned to the *MIDORI2* database. With the blocker, sparrow reads fell to a mean of 48% of taxonomically assigned reads, but nonsparrow passerine reads were essentially unchanged (Figure 2A). In effect, the improved sequencing power for target (i.e., nonsparrow animal) DNA increased median target read counts per nest by several reads (Figure 2B). However, despite more target reads in sparrow nests, mean species richness per nest was not significantly different with the blocker (mean 12.2, SD 6.9) than without (mean 12.1, SD 7.7); *t* (17) = −0.11, *p* = 0.91. Brewer's Sparrow nests had higher mean species richness per nest with the blocker (mean 13.1, SD 9.1) than without (mean 11, SD 9.5), but the gain was nonsignificant; *t* (8) = −1.48, *p* = 0.18. We lacked adequate sample sizes for a proper statistical evaluation of blocker effects on the remaining sparrows; patterns suggest increased species richness in Green‐tailed Towhee nests but not those of other sparrows (Figure 2C,D, Figure S2). Results were very similar when using minimum relative read abundance thresholds rather than minimum read thresholds (Figure S3) and we here employ the latter for simplicity. Site‐wide species richness increased from 82 to 87 (6%) in sparrow nests when the blocker was used. Gains were increasingly pronounced when applying minimum read thresholds: 78 versus 86 species (10% boost) when ≥ 2 reads were required, 58 versus 66 (14% boost) when ≥ 5 reads were required, and 36 versus 47 (31% boost) when ≥ 10 reads were required (Figure S4).

**FIGURE 3:**
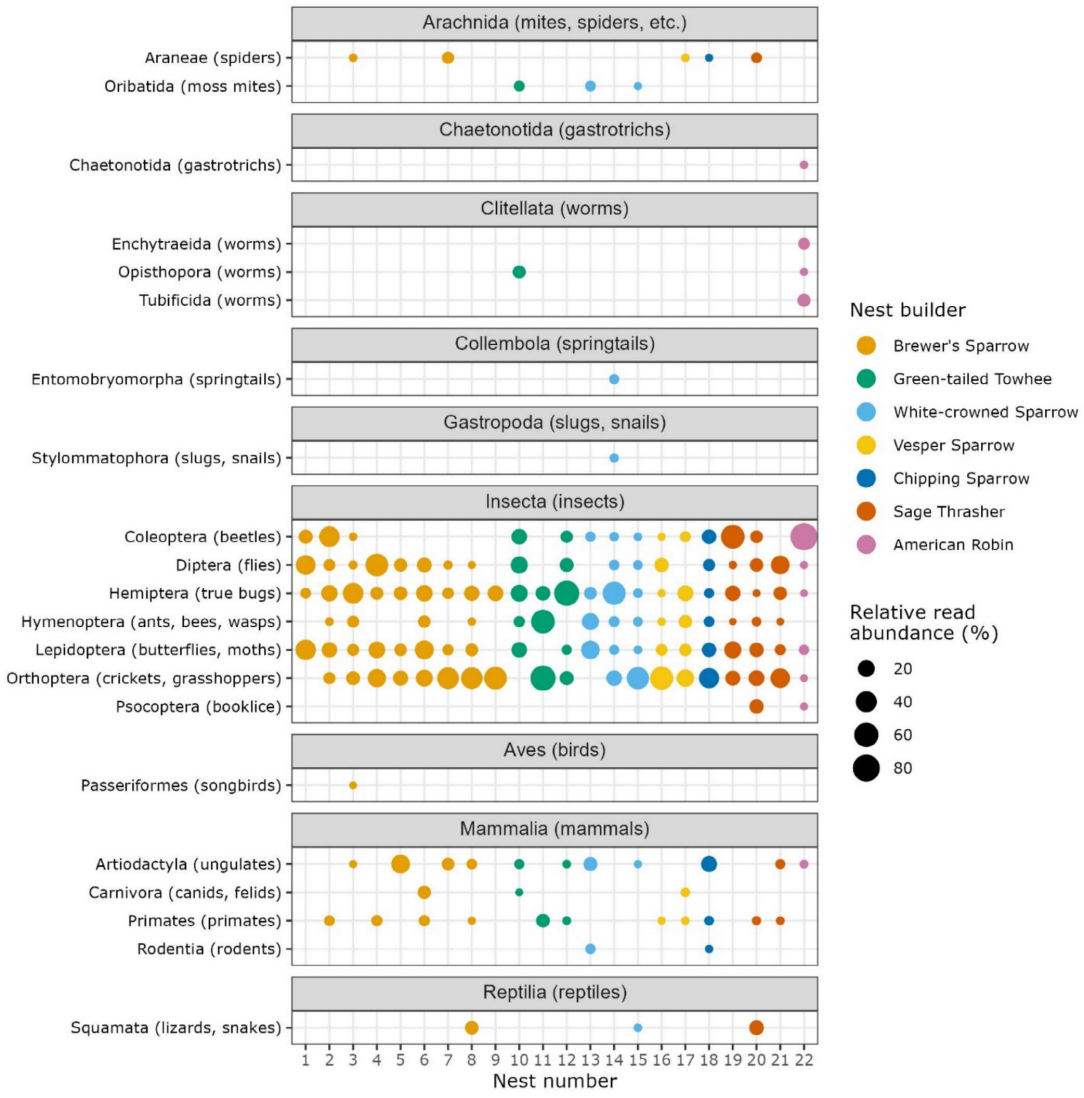
Proportion of reads per nest assigned to each order, excluding nest building songbirds and grouped by class. Data produced with and without the sparrow blocker were pooled for this analysis, and no minimum read threshold was used.

**FIGURE 4:**
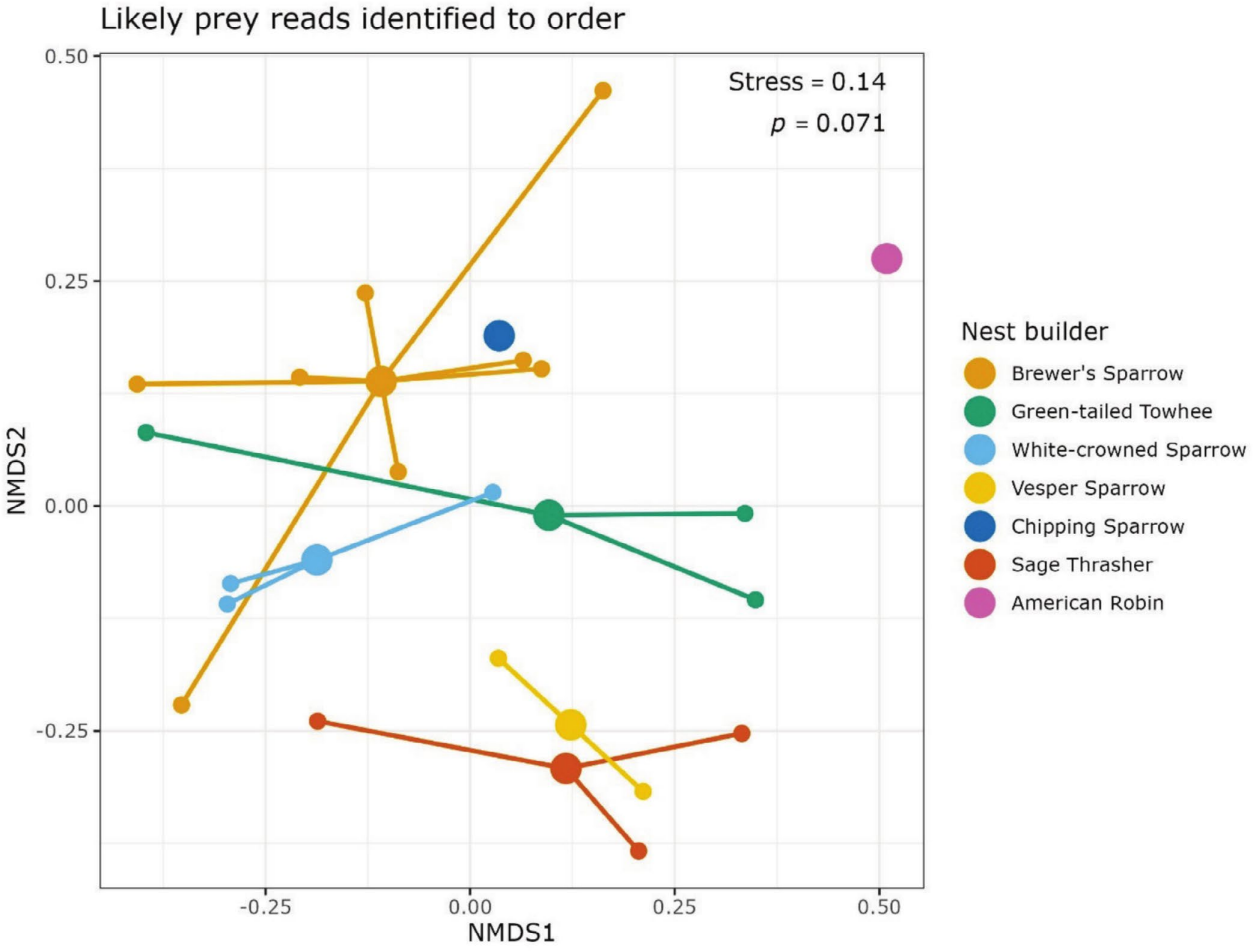
Nonmetric multidimensional scaling ordination of nests (small circles) and nest centroids (large circles) based on the number of reads from each ASV within likely prey orders. Data produced with and without the sparrow blocker were pooled for this analysis, and no minimum read threshold was used.

**FIGURE 5:**
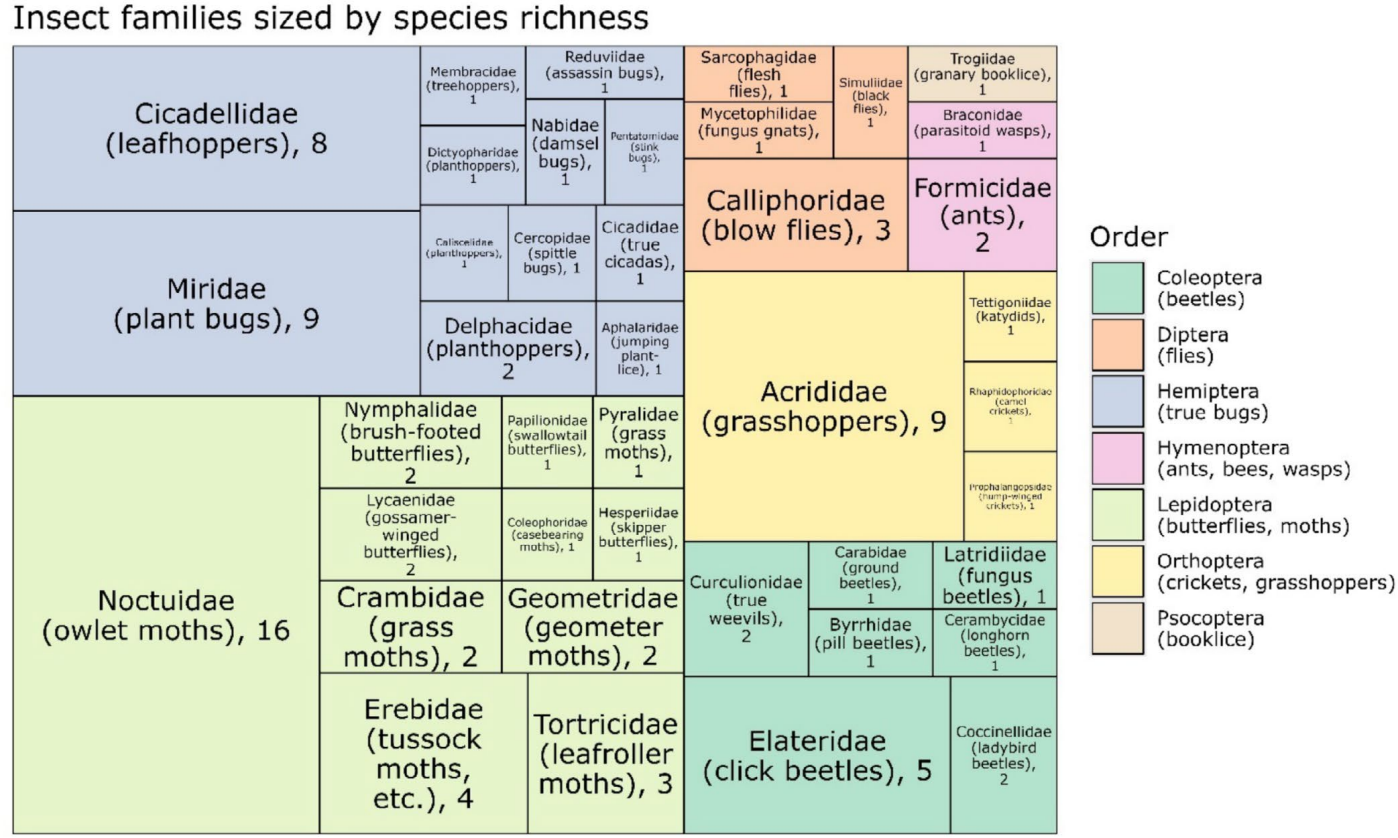
Treemap of all insect families detected, grouped by order and sized by species richness, with the number of distinct species listed after family names. Data produced with and without the sparrow blocker were pooled for this analysis and no minimum read threshold was used. All species feasibly occur at the study site.

Species detected with at least one read (*n* = 126) spanned 21 orders in 9 classes: Insecta (insects; *n* = 98 species), Aves (birds; *n* = 8), Mammalia (mammals; *n* = 6), Arachnida (mites, spiders, etc.; *n* = 5), Clitellata (worms; *n* = 4), Chaetonotida (gastrotrichs; *n* = 2), Collembola (springtails; *n* = 1), Gastropoda (slugs, snails; *n* = 1), and Reptilia (reptiles; *n* = 1) (Figure 3). All species were confirmed to feasibly occur at the study site. Differences in read counts from ASVs representing likely prey orders segregated nest‐building songbirds when ordinated via nonmetric multidimensional scaling, although the overall effect was nonsignificant (stress = 0.14, *p* = 0.071) (Figure 4). The 98 total insect species detected spanned 41 families, primarily Noctuidae (owlet moths; *n* = 16 species), Acrididae (grasshoppers; *n* = 9), Miridae (plant bugs; *n* = 9), and Cicadellidae (leafhoppers; *n* = 8) (Figure 5). Species accumulation curves increased linearly with nest number and did not asymptote, indicating that metabarcoding of additional nests would likely continue to yield new detections at a similarly high rate (Figure S5).

Notable detections include ectoparasitic blowflies of nestling birds ( *Protocalliphora sialia* and *Trypocalliphora braueri*) in 18 (82%) of 22 total nests. These were commonly observed in the field while nest monitoring but had not been identified to species. We successfully detected predator DNA in 8 (36%) of 22 nests, including gartersnake (*Thamnophis* sp.; *n* = 3 nests), long‐tailed weasel ( *Mustela frenata* ; *n* = 3), and yellow‐pine chipmunk ( *Tamias amoenus* ; *n* = 2). Predator identity was suspected from field observation or video monitoring for six of these nests, and eDNA detections matched suspected identity for five nests. The one prediction that did not match expectations—chipmunk detected but weasel suspected—was low confidence as a weasel was only observed in the general area, not at the nest. All other suspected predators, which were not detected, were raptors. See Data S1 for sample metadata including videos of predation events, *in silico* testing results demonstrating primer generality and blocker specificity, and read counts with taxonomic assignments.

## 4 Discussion

We have expanded upon an initial proof‐of‐concept by Levesque‐Beaudin et al. (2023) that eDNA metabarcoding of songbird nests is practical. While they extracted eDNA from three nest components separately, we demonstrated an efficient whole‐nest eDNA extraction technique. We also evaluated the information gained by inhibiting amplification of nest‐building songbird templates and, more generally, tested the performance of a blocking oligonucleotide design strategy presented by Prout et al. (2023) that utilizes alternating locked nucleic acids (LNAs) to minimize coblocking of species of interest.

It is difficult to compare blocker performance across studies given the many contributing factors, such as specialized chemistry (e.g., LNAs, peptide nucleic acids, inosine runs for dual priming), mechanism of action (annealing inhibition or elongation arrest), and absolute and relative concentrations of blockers and primers (Homma et al. 2022; Piñol et al. 2015; Prout et al. 2023; Robeson et al. 2018; Rojahn et al. 2021; Tan and Liu 2018). Still, our short (16 bp) elongation arrest blocker with alternating locked nucleic acids (LNAs) and mismatches concentrated towards the center for maximum impact performed extremely well, with ~100% blocking efficiency for New World sparrows and no discernible coblocking effects. For example, nonsparrow nest builders mismatched the blocker at either three sites total and one in the center (Sage Thrasher) or four sites total and two in the center (American Robin) (Data S1). When the sparrow blocker was used, read counts for these species stayed the same or even slightly increased (Figure 2A). This aligns with the finding by Prout et al. (2023) that with alternating LNAs, a single center mismatch is enough to strongly limit blocking (retaining ~90% of reads in their study). One consequence for our blocker is that it will not work well for 25% of the New World sparrows we tested *in silico*, which have one or more mismatches. We therefore recommend ensuring a perfect match with the sparrow of interest prior to use.

High blocker efficiency and specificity paid off by increasing target read counts (Figure 2B) and the overall number of species detected (Figures S4 and S5). However, blocker impacts on species richness were inconsistent among sparrow species and were negative overall for Vesper and Chipping Sparrow (Figure 2C,D). This appears to have resulted from considerable stochasticity between nests (Figure S2). Indeed, even Brewer's Sparrow and Green‐tailed Towhee, with positive blocker effects on average, had neutral or negative blocker effects in some cases. Such variability highlights the value of PCR replication. We pooled four PCR replicates per nest and blocker analysis, which is low enough to miss rare species (Ficetola et al. 2015; Shirazi et al. 2021). Higher technical replication would likely have resulted in more consistent blocker effects across sparrows and enabled us to draw stronger conclusions.

As is common when metabarcoding environmental samples, the majority of reads were in fact nontarget (i.e., nonanimal). In our study, the large majority were fungal and likely usurped much of the sequencing power that would have otherwise been applied to nonsparrow animal species. Indeed, assigning taxonomy to the top BLAST hit for each ASV revealed that, without the sparrow blocker, fungi produced 57% of reads in sparrow nests, versus 29% for sparrows and 4% for nonsparrow animals. With the blocker, this changed to 81%, ~0%, and 5%, respectively (Figure S1). Nonsparrow animal reads increased by relatively little compared to fungal reads. Future metabarcoding studies focused on nests and other environmental samples may benefit from more specific primers or additional blockers targeting fungi, or whichever ASVs are identified in pilot studies to constitute the majority of “background” reads. For example, we used a single, universal animal primer pair to inform all study objectives (determination of nestling diet, parasites, and predators), but unique primers for each objective (e.g., targeting arthropods for diet and parasites and vertebrates for predators) may have afforded greater detection sensitivity. Additional markers, though, would likely increase costs and reduce sequencing depth per amplicon, making blockers appealing when particularly abundant off‐target taxa can be identified. Alternative to metabarcoding, the highest sensitivity of all can likely be achieved through species‐specific amplification followed by next‐generation sequencing (McCarthy et al. 2023) or, with the inclusion of a hydrolysis probe, quantitative PCR (qPCR) or droplet digital PCR (ddPCR) (McColl‐Gausden et al. 2023; Wood et al. 2019). Downsides of these approaches include: (1) the suite of taxa of interest must be identified beforehand, (2) more up‐front marker development may be required, and (3) the number of taxa capable of being screened prior to eDNA sample exhaustion is limited. If very high sensitivity is required for more than a few species (up to several dozen), high‐throughput qPCR may be the most appropriate option (Elmore et al. 2025; Kronenberger et al. 2025; Sleeting et al. 2025).

The biodiversity we resolved offers a rare glimpse into avian trophic interactions. There was a good deal of internest variability in our study, but overall detections aligned with expected nestling diets (Figures 3, 4, 5) (Petersen and Best 1986; Howe et al. 2000) and prior arthropod surveys in sagebrush and coniferous ecosystems (Sanford and Huntly 2010; Haab et al. 2023; Christiansen et al. 1989). While we focused on primarily insectivorous species in this study, we note that (with appropriate primer selection) our methods could be particularly useful for identifying more cryptic nestling diets such as those of frugivores that regurgitate food directly into nestling mouths; such information would have strong applications for elucidating fruit‐frugivore networks, for example. Detection of ectoparasitic blowflies in 82% of nests corroborated observations of common blowfly presence during nest monitoring, primarily as larvae on or in the skin of nestlings. Their prevalence is significant given previously documented negative effects on nestling condition and fledgling survival (Streby et al. 2009), and the potential for some taxa to act as vectors for avian influenza (Fujita et al. 2024). Detection of nest predators was moderately successful but was inconsistent and mostly occurred with single‐digit read counts. Notably, we did not detect predatory raptors (0/5 nests) and detected a lower proportion of snakes than mammals (2/7 and 4/5 nests, respectively; predator identity was uncorroborated for the remaining two nests with detections). This is likely due to a combination of brief nest contact from raptors (mean of 8 s per nest) compared with mammals and snakes (means of 105 s and 1810 s, respectively) and potentially lower DNA shedding rates for snakes (Nordstrom et al. 2022).

Songbird nest metabarcoding, as demonstrated in this study, is capable of providing novel insights into avian ecology (Figures 3 and 4) and general biodiversity, particularly for insects (Figure 5). Such data can bolster the efficiency of currently laborious and taxonomically coarse investigations into avian trophic interactions. An important caveat, though, for diet studies is the inability to fully equate species detection with species consumption. Detections may represent contamination in the laboratory, artifacts from index switching or sequencing errors, or misidentifications in reference databases. When false positives are of particular concern, practitioners may consider applying higher minimum read thresholds, potentially informed by concurrent sequencing of negative controls (Drake et al. 2021). Even in lieu of false positives, species detected may not actually represent diet items, but interactions with nest material prior to nest building, nest occupants, predators of nest occupants, or even prey from another trophic level (e.g., consumed by a spider that was consumed by a bird). Another important consideration is songbird foraging behavior; detections in nests of songbirds that range widely when foraging are more difficult to attribute to species presence at the nest location. This may not be an issue for songbirds with smaller home ranges and higher territoriality. For example, in this same system, Zarri et al. (2024) found mean territory size to be relatively small, ranging from 0.49 ha for Brewer's Sparrows to 4.86 ha for Sage Thrashers. Breeding songbirds typically forage within their territories and therefore presumably aggregate exclusively local eDNA, though what constitutes “local” will vary by species (Wiens et al. 1985). It is worth noting that this issue is not specific to nest metabarcoding: many other eDNA substrates like water, air, and scat collate biodiversity information from across broad geographic areas, and detections must be interpreted accordingly. Nest metabarcoding generates such rich and unique data that we believe its utility far outweighs potential ambiguities in results. With broader uptake, nest metabarcoding has the capacity to complement methods across the fields of ecology and conservation biology.

## Author Contributions

**John A. Kronenberger:** conceptualization (equal), data curation (lead), formal analysis (lead), investigation (lead), methodology (lead), resources (equal), validation (lead), visualization (lead), writing – original draft (lead), writing – review and editing (equal). **Elise C. Zarri:** conceptualization (equal), data curation (supporting), formal analysis (supporting), investigation (supporting), methodology (supporting), resources (equal), writing – original draft (supporting), writing – review and editing (equal). **Anna Noson:** conceptualization (supporting), funding acquisition (lead), investigation (supporting), resources (equal), writing – review and editing (equal). **Taylor M. Wilcox:** conceptualization (supporting), investigation (supporting), methodology (supporting), resources (equal), writing – review and editing (equal).

## Conflicts of Interest

The authors declare no conflicts of interest.

## Supporting information


**Data S1:** ece372164‐sup‐0001‐DataS1.xlsx.

**Figure S1:** Relative read abundance (RRA) of ASVs assigned to kingdom according to the closest BLAST hit, with and without the sparrow blocker. One Brewer's Sparrow nest is excluded from this analysis due to unusually low read counts without the sparrow blocker; this was attributed due to an indexing error.
**Figure S2:** Impacts of the sparrow blocker on the number of species detected per nest for a range of minimum read thresholds. Note that one Brewer's Sparrow nest (MT.21.GAA.04) had unusually low read abundance without the sparrow blocker, attributed to an indexing issue; this nest was excluded from the mean species richness curves in the main text.
**Figure S3:** Impacts of the sparrow blocker on the mean number of species detected per nest for a range of minimum relative read abundance (RRA) thresholds (A) and percent change in species richness when the blocker was used, with the dotted line at 0 indicating no difference (B). All nest builders are sparrows except for Sage Thrasher and American Robin. The number of nests per species varies: Brewer's Sparrow, *n* = 8 (one excluded due to unusually low read abundance without the sparrow blocker, attributed to an indexing issue); Green‐tailed Towhee, *n* = 3; White‐crowned Sparrow, *n* = 3; Vesper Sparrow, *n* = 2; Chipping Sparrow, *n* = 1; Sage Thrasher, *n* = 3; American Robin, *n* = 1.
**Figure S4:** Waffle plots illustrating the increase in species richness (organized by class) in sparrow nests with the sparrow blocker for a range of site‐wide minimum read thresholds: no threshold, species with only singletons removed, species with < 5 reads removed, and species with < 10 reads removed. Numbers at the top right of each plot indicate species richness.
**Figure S5:** Species accumulation curves produced through 100 random permutations of the data (subsampling without replacement), separated by analysis: with and without the sparrow blocker and all data pooled.

## Data Availability

Supporting data are in a supplemental spreadsheet, including nest metadata, GenBank accession numbers, and results from *in silico* primer and blocker testing, and read counts and taxonomic assignments. Videos of nest predation events can be accessed at https://data.mendeley.com/datasets/stw9c65kc3/1.
