# Supplementary material for: Metabarcoding Close to Home: Songbird Nests as eDNA Aggregators for Trophic Ecology and Biodiversity Studies
Source: Ecol Evol. 2025 Oct 13;15(10):e72164. doi: 10.1002/ece3.72164 (PMC12518783; doi:10.1002/ece3.72164)
Supplement: Supplementary file 2 — Figure S1: Relative read abundance (RRA) of ASVs assigned to kingdom according to the closest BLAST hit, with and without the sparrow blocker. One Brewer's Sparrow nest is excluded from this analysis due to unusually low read counts without the sparrow blocker; this was attributed due to an indexing error. Figure S2: Impacts of the sparrow blocker on the number of species detected per nest for a range of minimum read thresholds. Note that one Brewer's Sparrow nest (MT.21.GAA.04) had unusually low read abundance without the sparrow blocker, attributed to an indexing issue; this nest was excluded from the mean species richness curves in the main text. Figure S3: Impacts of the sparrow blocker on the mean number of species detected per nest for a range of minimum relative read abundance (RRA) thresholds (A) and percent change in species richness when the blocker was used, with the dotted line at 0 indicating no difference (B). All nest builders are sparrows except for Sage Thrasher and American Robin. The number of nests per species varies: Brewer's Sparrow, n = 8 (one excluded due to unusually low read abundance without the sparrow blocker, attributed to an indexing issue); Green‐tailed Towhee, n = 3; White‐crowned Sparrow, n = 3; Vesper Sparrow, n = 2; Chipping Sparrow, n = 1; Sage Thrasher, n = 3; American Robin, n = 1. Figure S4: Waffle plots illustrating the increase in species richness (organized by class) in sparrow nests with the sparrow blocker for a range of site‐wide minimum read thresholds: no threshold, species with only singletons removed, species with < 5 reads removed, and species with < 10 reads removed. Numbers at the top right of each plot indicate species richness. Figure S5: Species accumulation curves produced through 100 random permutations of the data (subsampling without replacement), separated by analysis: with and without the sparrow blocker and all data pooled. [file ECE3-15-e72164-s002.docx]

Metabarcoding close to home: Songbird nests as eDNA aggregators for trophic ecology and biodiversity studies

John A. Kronenberger, Elise C. Zarri, Anna Noson, and Taylor M. Wilcox

**SUPPLEMENTARY FIGURES**


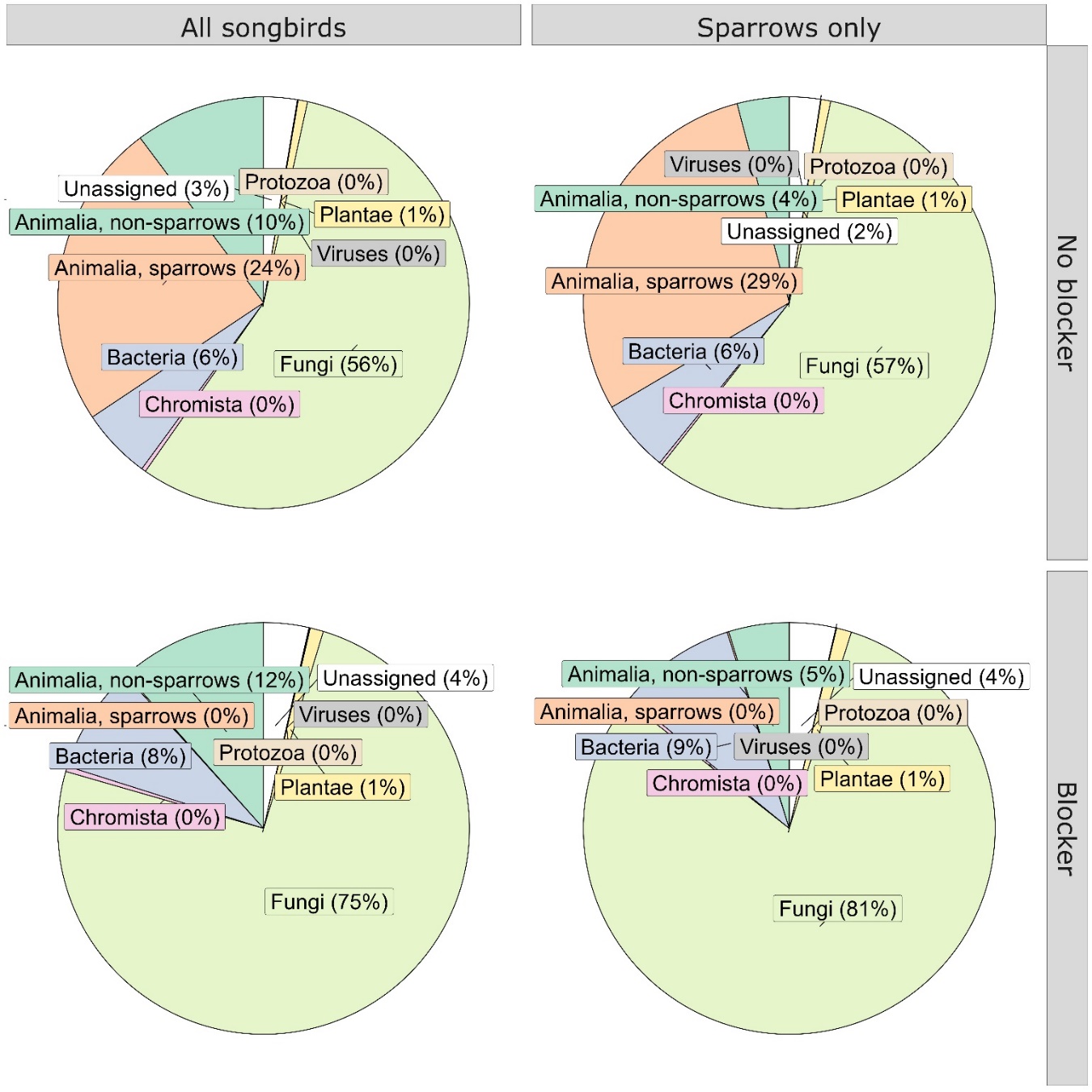


**Figure S1**: Relative read abundance (RRA) of ASVs assigned to kingdom according to the closest BLAST hit, with and without the sparrow blocker. One Brewer's Sparrow nest is excluded from this analysis due to unusually low read counts without the sparrow blocker; this was attributed due to an indexing error.


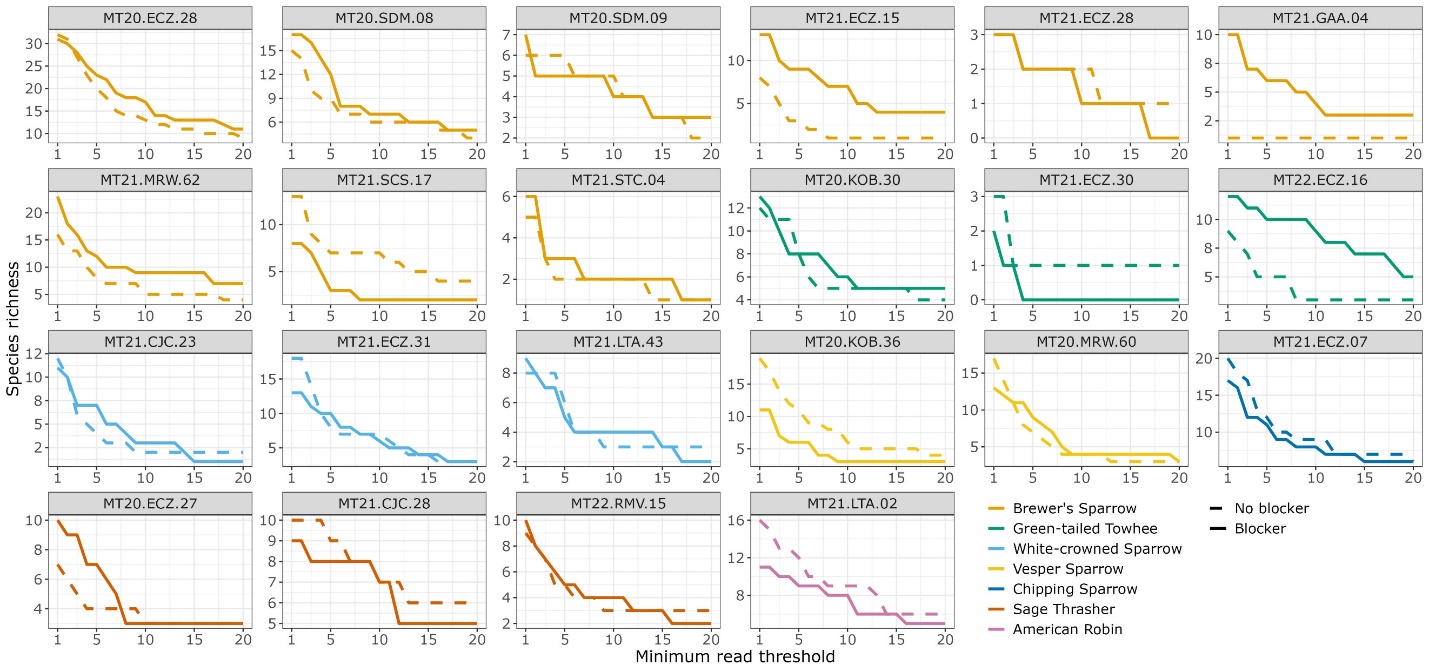


**Figure S2**: Impacts of the sparrow blocker on the number of species detected per nest for a range of minimum read thresholds. Note that one Brewer's Sparrow nest (MT.21.GAA.04) had unusually low read abundance without the sparrow blocker, attributed to an indexing issue; this nest was excluded from the mean species richness curves in the main text.


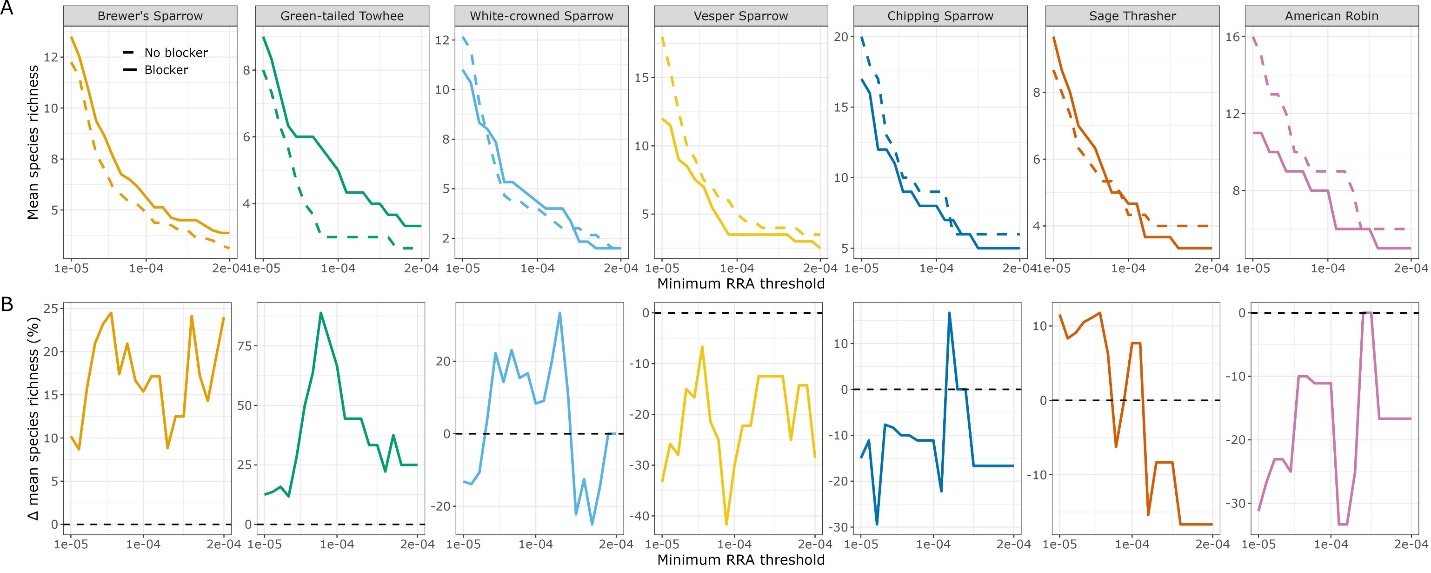


**Figure S3**: Impacts of the sparrow blocker on the mean number of species detected per nest for a range of minimum relative read abundance (RRA) thresholds (A) and percent change in species richness when the blocker was used, with the dotted line at 0 indicating no difference (B). All nest builders are sparrows except for Sage Thrasher and American Robin. The number of nests per species varies: Brewer’s Sparrow, n = 8 (one excluded due to unusually low read abundance without the sparrow blocker, attributed to an indexing issue); Green-tailed Towhee, n = 3; White-crowned Sparrow, n = 3; Vesper Sparrow, n = 2; Chipping Sparrow, n = 1; Sage Thrasher, n = 3; American Robin, n = 1.


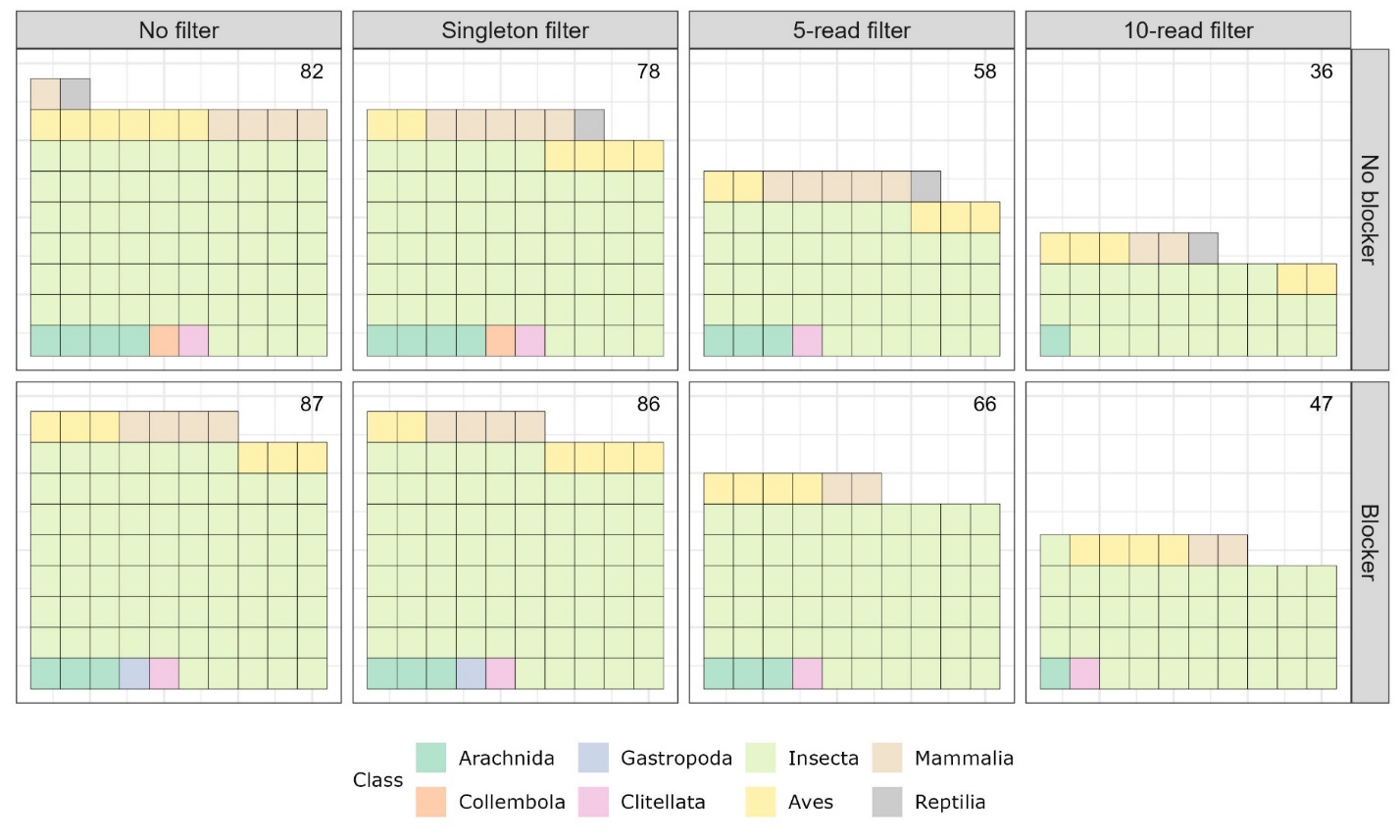


**Figure S4**: Waffle plots illustrating the increase in species richness (organized by class) in sparrow nests with the sparrow blocker for a range of site-wide minimum read thresholds: no threshold, species with only singletons removed, species with < 5 reads removed, and species with < 10 reads removed. Numbers at the top right of each plot indicate species richness.


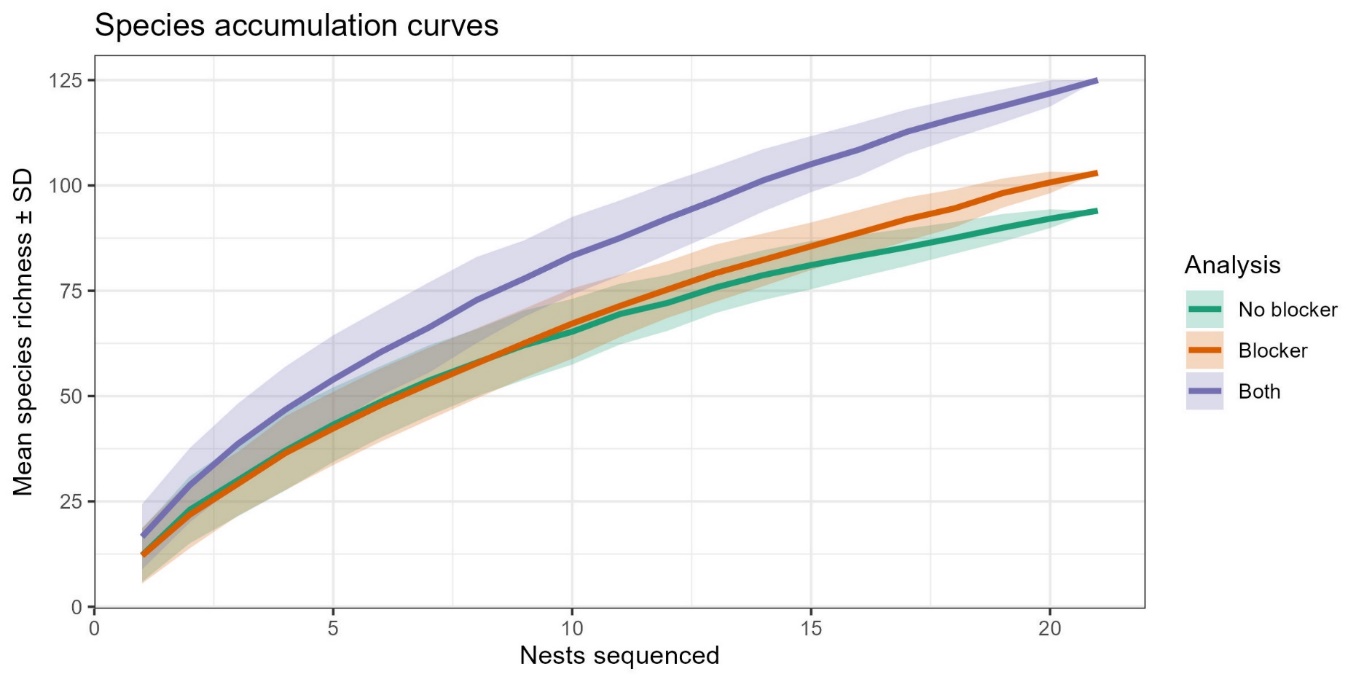
**Figure S5**: Species accumulation curves produced through 100 random permutations of the data (subsampling without replacement), separated by analysis: with and without the sparrow blocker and all data pooled.
